# Improved subcutaneous edema segmentation on abdominal CT using a generated adipose tissue density prior

**DOI:** 10.1007/s11548-023-03051-5

**Published:** 2024-01-17

**Authors:** Jianfei Liu, Omid Shafaat, Sayantan Bhadra, Christopher Parnell, Ayden Harris, Ronald M. Summers

**Affiliations:** 1 Imaging Biomarkers and Computer-Aided Diagnosis Laboratory, Clinical Center, National Institutes of Health, Bethesda, MD 20892 USA; 2 Diagnostic Radiology, Walter Reed National Military Medical Center, Bethesda, MD 20889 USA

**Keywords:** Generative adversarial network, Level set segmentation, Adipose tissue, Edema segmentation

## Abstract

**Purpose:**

Edema, or swelling, is a common symptom of kidney, heart, and liver disease. Volumetric edema measurement is potentially clinically useful. Edema can occur in various tissues. This work focuses on segmentation and volume measurement of one common site, subcutaneous adipose tissue.

**Methods:**

The density distributions of edema and subcutaneous adipose tissue are represented as a two-class Gaussian mixture model (GMM). In previous work, edema regions were segmented by selecting voxels with density values within the edema density distribution. This work improves upon the prior work by generating an adipose tissue mask without edema through a conditional generative adversarial network. The density distribution of the generated mask was imported into a Chan-Vese level set framework. Edema and subcutaneous adipose tissue are separated by iteratively updating their respective density distributions.

**Results:**

Validation results on 25 patients with edema showed that the segmentation accuracy significantly improved. Compared to GMM, the average Dice Similarity Coefficient increased from 56.0 to 61.7% ($p<0.05$) and the relative volume difference decreased from 36.5 to 30.2% ($p<0.05$).

**Conclusion:**

The generated adipose tissue density prior improved edema segmentation accuracy. Accurate edema volume measurement may prove clinically useful.

## Introduction

Edema is swelling that results in fluid retention [1]. It often occurs within subcutaneous tissue [2]. Its presence could be caused by kidney disease [3], heart failure [4], and liver cirrhosis [5, 6]. There are currently several ways edema is assessed in the clinic. For example, edema can be graded by physically pressing on edematous tissue (e.g., the leg or body wall) and assessing the pit depth and recovery time [1]. Another method assesses sodium homeostasis using body weight and other factors using the Edelman equation [7, 8]. Both measurements are also useful to assess patients with an exacerbation of heart failure [9] and monitor the nutrient intake of older recuperative care patients [10]. However, such grading can only approximately measure the amount of edema fluid. Another approach is to assess body weight change although this measurement sometimes has to be adjusted, such as for severely burned patients [11]. Direct volume measurement of edema using imaging could be a useful supplement to evaluate these conditions.

**Fig. 1:**
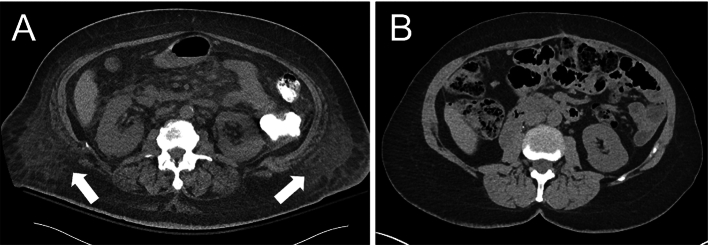
Comparison of patients without and with edema on abdominal CT. Edema is visually represented as heterogeneous regions within the adipose tissue (arrows, **A**). In contrast, adipose tissue is homogenous in a patient without edema (**B**)

Automatic edema measurement is nontrivial. Rather than being localized like a tumor or an organ, edema is usually heterogeneous, discontinuous and highly variable in distribution and shape (Fig. 1). For this reason, there is little research on edema segmentation. In previous work [12], a two-class Gaussian mixture model was developed to represent CT density distributions of edema and subcutaneous adipose tissue. Edema regions were segmented by identifying voxels within the density values within the edema density distribution.

Adipose tissue segmentation based on deep learning has attracted much attention in the past decade [13–17]. They are generally categorized into 2D [13, 14] and 3D [15–17] U-Net variants. These works rely on manual annotations of adipose tissue for training deep learning models. However, it is difficult to manually annotate edema because the edema is diffused within the adipose tissue with unclear boundaries (Fig. 1). That is the major difficulty for applying deep learning to edema segmentation [12].

Expanding upon the idea of unsupervised density distribution modeling [12], this paper integrates this modeling into a Chan-Vese level set segmentation framework [18]. The density distributions of edema and adipose tissue are iteratively updated with level set propagation. In addition, the density histogram is computed from an adipose tissue mask created by a conditional generative adversarial network (C-GAN) [19], which is used as the tissue density prior. Comparing the histogram of the density prior and the one from the adipose tissue regions in the CT image leads to an additional constraint for level set propagation, which helps achieve better segmentation accuracy.

## Methods

### Adipose tissue density prior from C-GAN

**Fig. 2:**
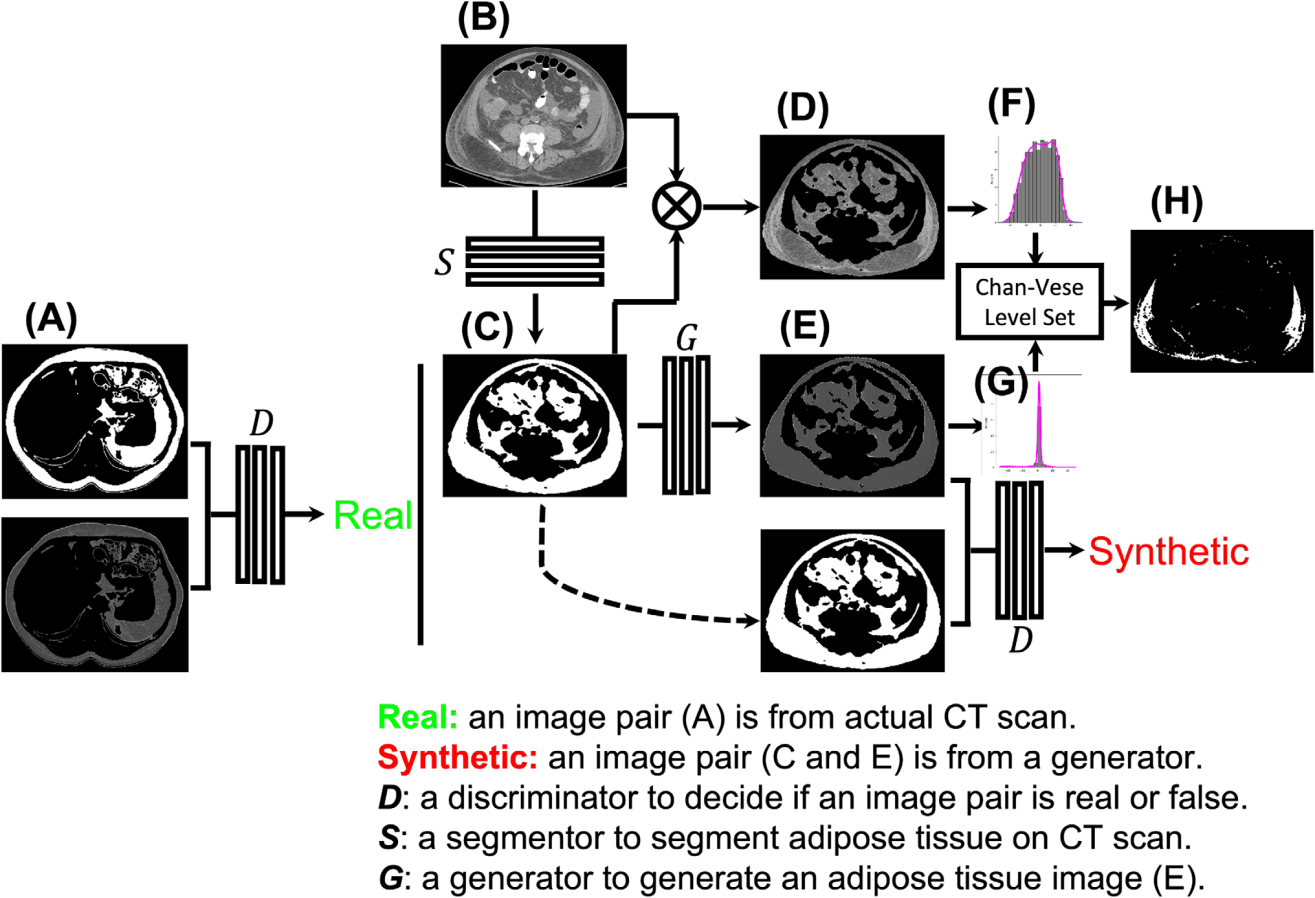
Processing pipeline for edema segmentation on CT images. A C-GAN consisting of a discriminator *D* and a generator *G* is trained beforehand on paired, 2D binary and grayscale adipose tissue masks (**A**) from patients without edema. During edema segmentation, a CT image (**B**) of an edema patient is imported into a segmentor *S* to obtain its adipose tissue segmentation mask (**C**). Multiplying **B** and **C** yields a non-synthetic grayscale adipose tissue mask (**D**). Importing **C** into the generator *G* also generates a synthetic grayscale tissue mask without edema (**E**). The adipose tissue density histogram (**G**) is constructed from (**E**), which is embedded into the level set segmentation framework by comparing it with the one (**F**) from (**D**). Propagating level set yields the final edema segmentation (**H**)

The key contribution of this work is to generate an adipose tissue mask and better tissue density prior to differentiate adipose tissue from edema. C-GAN [19] is used because it can artificially generate original images from binary masks. Similar to the original C-GAN [19], PatchGAN and U-Net are used to formulate the discriminator *D* and the generator *G*, respectively. Unlike the original C-GAN that uses paired original images and binary masks as input, a grayscale image mask (bottom image, Fig. 2A) is chosen to replace the original image by only keeping adipose tissue regions in a CT image. Paired binary and grayscale masks from patients without edema (Fig. 2A) are used to train the C-GAN.

Once C-GAN is trained, it is used to generate a grayscale adipose tissue mask without edema (Fig. 2E) for a CT image with edema (Fig. 2B). The image is input into a segmentor *S* (Fig. 2) to get its corresponding adipose tissue segmentation (Fig. 2C). Here, *S* is a residual U-Net [17]. Assuming the background value is zero in the segmentation mask (Fig. 2C), multiplying it with the CT image (Fig. 2B) leads to a non-synthetic grayscale adipose tissue mask with edema (Fig. 2D), $I_{ns}(\textbf{x}), \textbf{x}\in \Omega$, where $\Omega$ is the image domain. Importing the segmentation mask (Fig. 2C) into the C-GAN also generates a synthetic grayscale tissue mask without edema (Fig. 2E), $I_s(\textbf{x})$, as the density prior. Two histograms, $G(I_{ns})$ and the $G(I_s)$, are constructed. The normalized correlation of the two histograms is used as the addition constraint for edema segmentation

1 $$\begin{aligned} F(I_{ns}, I_s) =\frac{\sum _\Omega \left( G(I_{ns})-\bar{G}(I_{ns}) \right) \left( G(I_s)-\bar{G}(I_s) \right) }{\sqrt{\sum _\Omega \left( G(I_{ns})-\bar{G}(I_{ns}) \right) ^2\sum _\Omega \left( G(I_s)-\bar{G}(I_s) \right) ^2}} \nonumber \\ \end{aligned}$$

Here, $\bar{G}=\sum _\Omega G/N$ and *N* is the total number of histogram bins.

### Chan-vese level set edema segmentation

The next step is to use the Chan-Vese level set framework [18] to segment edema with the additional constraint of Eq. 1. Let $\phi : \Omega \rightarrow \mathbb {R}$ be a signed distance function that represents the level set function [20].

2 $$\begin{aligned} \begin{aligned} E&= \int _\Omega \{\left( I_{ns}(\textbf{x})-\textsf{C}_1\right) ^2H(\phi )+\left[ \left( I_{ns}(\textbf{x})-\textsf{C}_2\right) ^2 \right. \\&\quad \left. +\alpha F(I_{ns}, I_s)\right] (1-H(\phi ))+\beta \vert \nabla H(\phi )\vert \}d\textbf{x} \end{aligned} \end{aligned}$$

Here, *H*(*x*) is the Heaviside function, with $H(\phi )=1$ if $\phi \ge 0$; otherwise $H(\phi )=0$. $\textsf{C}_1$ and $\textsf{C}_2$ are mean values of the edema and adipose tissue regions inside and outside of the level set $\phi$. $\int _\Omega \vert \nabla H(\phi )\vert d\textbf{x}$ represents the length of $\phi$. $\alpha$ and $\beta$ are hyperparameters that balance the influence of adipose density prior and the length of $\phi$, respectively. Note that $F(I_{ns}, I_s)$ is only computed in the background regions of $\phi <0$ because they correspond to adipose tissue regions. Since the histogram $G(I_s)$ in Fig. 2G is a Gaussian, the generated adipose tissue is homogenous. $F(I_{ns}, I_s)$ in Eq. 1 will be maximized if an image region is diffused with edema because $G(I_{ns})$ is a two-class Gaussian mixture model. It will force the level set to continuously propagate and separate edema from adipose tissue. This process gradually splits the Gaussian mixture model into two Gaussian models. Eventually, $F(I_{ns}, I_s)$ will be minimized if an image region is composed of homogenous adipose tissue.

The minimization is solved by alternatively updating $\textsf{C}_1$, $\textsf{C}_2$, and $\phi$ [18]. For fixed $\phi$, the optimal values of $\textsf{C}_1$, $\textsf{C}_2$ are the mean values of edema and adipose tissue regions.

3 $$\begin{aligned} \textsf{C}_1&= \frac{\int _\Omega I_{ns}(\textbf{x})H(\phi )d\textbf{x}}{\int _\Omega H(\phi )d\textbf{x}} \end{aligned}$$

4 $$\begin{aligned} \textsf{C}_2&= \frac{\int _\Omega I_{ns}(\textbf{x})(1-H(\phi ))d\textbf{x}}{\int _\Omega (1-H(\phi ))d\textbf{x}} \end{aligned}$$

For the fixed $\textsf{C}_1$ and $\textsf{C}_2$, the level set evolution equation of $\phi$ is

5 $$\begin{aligned} \begin{aligned} \frac{\partial \phi }{\partial t}&=\delta (\phi )\left[ -(I_{ns}(\textbf{x}) - \textsf{C}_1)^2 + (I_{ns}(\textbf{x}) - \textsf{C}_2)^2\right. \\&\quad \left. +\alpha F(I_{ns}, I_s) +\beta \textrm{div}\left( \frac{\nabla \phi }{\vert \nabla \phi \vert }\right) \right] \end{aligned} \end{aligned}$$

Figure 2H shows the final edema segmentation computed using Eqs. 3–5 in the current CT image. Combining segmentation results of all images leads to the final edema segmentation on the current CT scan.

### CT data and validation methods

We used two datasets to train the adipose tissue generator and to validate edema segmentation accuracy, respectively. The first dataset consisted of 101 contrast-enhanced CT scans (52 females and 49 males, average age 66.64±5.12 years) from patients without edema. The majority of these scans were used for a previously published muscle segmentation paper [21]; this paper focuses on adipose tissue generation. One CT scan was removed due to the failure of creating adipose tissue masks.

The validation dataset consisted of 25 CT scans from 25 patients with edema (9 females and 16 males, average age 51.96±14.67 years). They were identified using a keyword search for “edema” and “anasarca” in radiology reports of CT scans that included the abdomen and pelvis. The presence of edema was confirmed with the guidance of an experienced radiologist. The exclusion criteria were scatter and significant motion artifacts. The demographics of these patients are presented in Table 1.

**Table 1:** Demographics of 25 patients with edema used for evaluating segmentation accuracy

Age (years)	Gender	Race	CT Types	Scanner
[22, 78]	16 Males	20 White	19 contrast	24 Siemens
51.96±14.67	9 Females	5 Black	6 non-contrast	1 Toshiba

A residual U-Net [17] was used to segment the adipose tissue on the 101 CT scans without edema. 6,403 paired 2D binary and grayscale tissue masks were randomly selected from the segmentation results to train C-GAN. For the CT scans with edema, five images with subcutaneous edema were randomly selected. The interval between adjacent slices was adjusted as large as possible to emphasize different body regions. ITK-SNAP software was used to manually annotate edema [22]. Each slice was first thresholded using a density range of $[-50, 50]$ HU. Adipose tissue segmentation masks [17] to remove tissues outside the subcutaneous region. Finally, the remaining thresholded results were manually edited by deleting non-edema and adding edema regions. The whole process was performed by a grader under the supervision of an experienced radiologist. Three additional graders (two radiology residents and one postdoctoral fellow) also preformed the same process to annotate 5 CT scans (5 slices per scan). Three sets of subcutaneous edema annotations were compared to understand the inter-observer difference.

The previous GMM method was chosen as the baseline [12]. We used four segmentation metrics to evaluate accuracy: intersection over union (IoU), Dice Similarity Coefficient (DSC), absolute volume difference (AVD) between manually annotated and automatically segmented edema, and relative volume difference (RVD), the ratio of AVD over manually-annotated edema volumes. Eighteen CT scans were contrast-enhanced, and the remaining ones were noncontrast-enhanced.

## Results

There was substantial variation of edema annotations among three graders, two residents in particular with IoU less than 50% (Table 2). One resident tended to include surrounding affected tissue as edema. It resulted in larger annotated regions than the ones from the other two graders.

**Table 2:** Annotation comparison between two radiology residents (A and B) and one postdoctoral fellow (C) on 5 abdominal CT scans with edema

Graders	IoU (%)	DSC (%)	AVD (liter)	RVD (%)
A vs. B	49.8±16.6	65.1±15.5	0.017±0.011	73.9±57.8
A vs. C	$51.3\pm 17.7$	$66.2\pm 16.7$	$0.016\pm 0.018$	$99.1\pm 106.8$
B vs. C	$74.2\pm 10.6$	$84.8\pm 7.1$	$0.003\pm 0.002$	$16.2\pm 16.8$

Across all four segmentation metrics, the proposed method significantly improved upon the baseline GMM ($p<0.05$) with IoU, DSC, and RVD values increasing by 5–6% and AVD decreasing by 0.003 ls (Table 3).

**Table 3:** Segmentation accuracy comparison between GMM [12] and the proposed method on 25 abdominal CT scans with edema

Method	IoU (%)	DSC (%)	AVD (liter)	RVD (%)
GMM	41.2±17.9	56.0±19.4	0.021±0.022	36.5±18.8
Proposed	$\mathbf {46.8\pm 17.5}$	$\mathbf {61.7\pm 17.7}$	$\mathbf {0.018\pm 0.020}$	$\mathbf {30.2\pm 16.4}$

Bold font indicates improvement

**Fig. 3:**
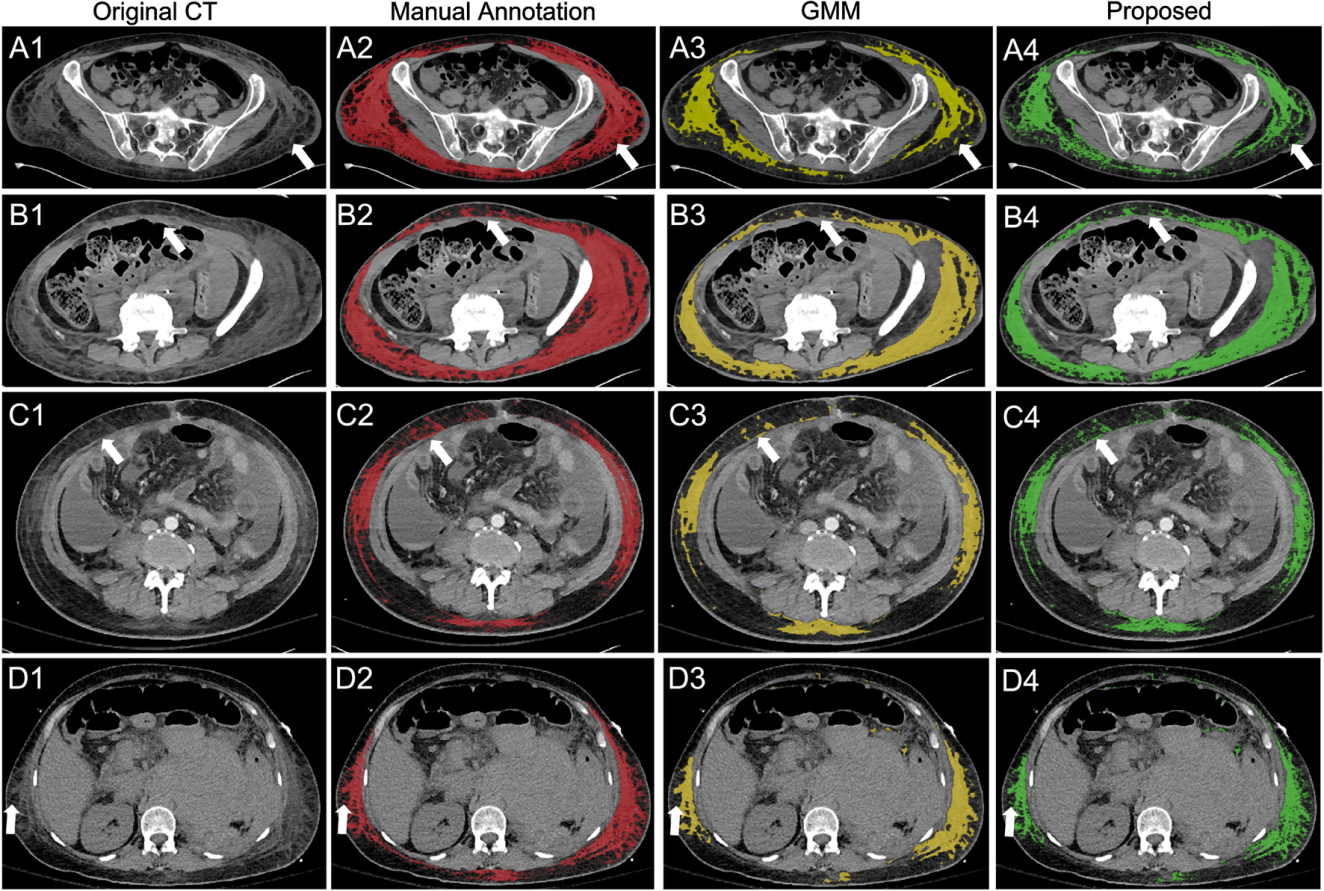
Edema segmentation results on four patients with edema. Each row (**A**–**D**) corresponds to a unique patient. Manual annotations of edema are shown in red (**A2**–**D2**), segmentations results from GMM are in yellow (**A3**–**D3**), and results from the proposed method are in green (**A4**–**D4**). Both GMM and the proposed methods extract main regions of edema well, but the proposed method preserves small, isolated edema (white arrows) better than GMM thanks to the adipose density prior constraint

Visual comparison of segmentation results on CT scans from four patients with edema also confirmed better segmentation accuracy with the proposed method (Fig. 3). Both GMM and the proposed method slightly under-segment edema in the pelvis in comparison with the manual annotation (top two rows). However, compared to GMM, the proposed method preserves small, isolated edema better (white arrows in Figs. 3A4 and 3B4). It is probably because the intensity prior of $F(I_{ns}, I_s)$ in Eq. 5 is high in these regions. It forces the level set to propagate till the edema and adipose tissue are separated. The under-segmentation issue was improved in the abdomen for both GMM and the proposed method (bottom two rows). However, the proposed method performed better on isolated edema.

## Conclusion and future work

In this paper, we developed a Chan-Vese level set framework with adipose tissue density prior for subcutaneous edema segmentation on abdominal CT. The key contribution lies in the use of a C-GAN to convert a binary adipose tissue mask to a grayscale one. Since C-GAN is trained on patients without edema, it generates edema-free adipose tissue masks even when the input binary masks are from patients with edema. In comparison with the baseline GMM method [12], the proposed method achieved better segmentation accuracy with 5–6% increase of IoU, DSC, and RVD and 0.003 ls decrease of AVD. Qualitatively, the proposed method preserved small, isolated edema foci better than did the GMM.

Under-segmentation of subcutaneous edema sometimes occurred in the pelvis. Additional priors, such as shape, are potentially useful to reduce the error. Segmentation results were only validated on 25 contrast enhanced CT scans with edema from a single institution. Future work will include more CT scans from different institution. The inter-observer study among two radiology residents and one postdoctoral fellow showed substantial variation on edema annotations, particularly between two residents. It demonstrated the importance of developing an automatic edema segmentation method, as it can measure edema volumes objectively. Visceral edema is not considered in this work although it is a useful indicator for some clinical scenarios [23–25]. The combined volume measurements of subcutaneous and visceral edemas could be useful for assessing patients with heart failure [9] and monitoring the nutrient intake of older recuperative care patients [10]. Moreover, ascites and pleural effusion are often observed in conjunction with edema in anasarca patients. Fortunately, our group developed a set of automatic tools to measure their volumes [26–28]. A list of fluid volume measurements would be more beneficial for clinical use. The integration of these tools with edema segmentation method is another direction in the future work.

Automatic segmentation of subcutaneous edema provides a good starting point to quantitatively evaluate conditions that produce edema. It could be a useful supplement to body weight measurements that are widely used in the clinic.
